# Hybrid and advanced convolution techniques for highly accurate, reliable and automated ECG classification

**DOI:** 10.1038/s41598-026-55382-3

**Published:** 2026-06-10

**Authors:** R. Gokul, Yaswanth Sree Penumalli, Chalamala Ranjith, Moilla Namitesh Reddy, D. S. Bhargava, G. Seetharaman, R. Dhanalakshmi, T. N. Prabakar, S. Devi Poonguzhali

**Affiliations:** 1 Department of ECE, School of EEE, SASTRA Deemed University, Thanjavur, Tamilnadu India; 2 Department of ECE, Indian Institute of Information Technology, Tiruchirappalli, Tamilnadu India

**Keywords:** ECG classification, 1D-CNN, Dynamic kernel switching, Self-attention, Dilated convolution, Focal loss, Cross-validation, Edge AI, Quantization, NPU, FPGA, MIT-BIH arrhythmia database, Cardiology, Computational biology and bioinformatics, Engineering, Mathematics and computing

## Abstract

Cardiac arrhythmias are a leading cause of cardiovascular mortality worldwide, and auto- mated analysis of electrocardiogram (ECG) recordings is an unmet clinical need. This paper introduces a hybrid 1D Convolutional Neural Network (CNN) architecture for automated ECG heartbeat classification that captures both multi-scale morphological and long-range temporal dependencies. The architecture combines Dynamic Kernel Switching (DKS) with adaptive gating over kernel sizes {3*,* 5*,* 7}, a Self-Attention module, and Dilated Convolutions at dilation rates 2 and 4. For low-power edge deployment on Neural Processing Units (NPUs) and FPGA/ASIC platforms, three quantization paths are evaluated: (1) Dynamic Post-Training Quantization (PTQ) with INT8 weights and FP32 I/O, (2) Full-Integer PTQ with INT8 weights and activations, and (3) Quantization-Aware Training (QAT). With a corrected stratified 72/8/20 train/validation/test protocol, focal loss (*γ* = 2*.*0), AdamW optimization and cosine learning rate decay, the proposed model achieves 98.99% test accuracy and a macro F1-score of 0.9484 on the MIT-BIH Arrhythmia Database (16,219 held-out test beats, five AAMI classes). Five-fold stratified cross-validation confirms statistical robustness: 98*.*93% ± 0*.*10% accuracy with 95% confidence interval [98.84%, 99.02%]. A comprehensive seven-variant ablation study covering Self-Attention, CBAM, CSA, and SimSA demonstrates that the proposed DKS + Self-Attention combination outperforms all alternatives. Generalization is further validated on five independently withheld MIT-BIH patient recordings (10,444 beats), achieving 92.86% accuracy and macro F1 of 0.8219. The model comprises only 331,733 parameters and reduces to ≈0*.*32 MB under Dynamic PTQ, enabling direct TFLite/INT8 conversion for RTL-level FPGA deployment.

## Introduction

**Fig. 1:**
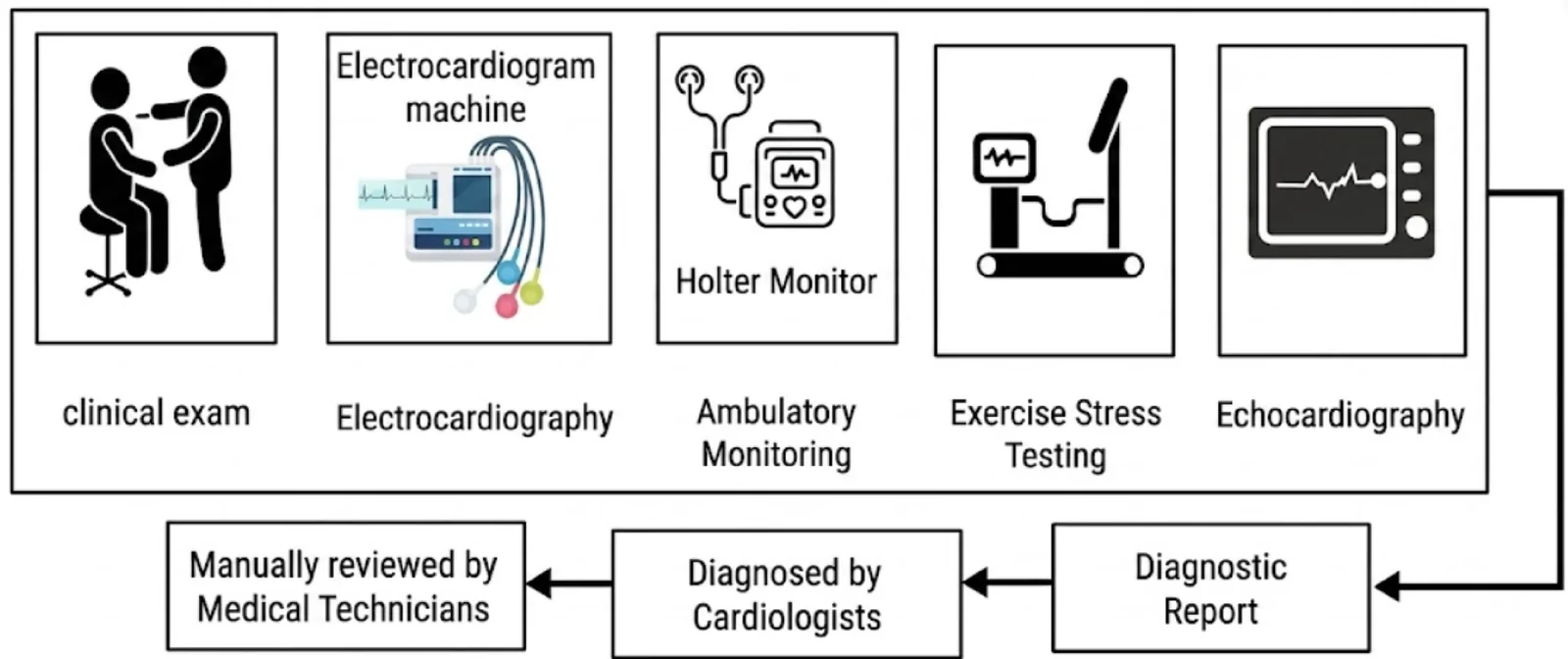
Traditional investigation for arrhythmia.

Arrhythmias irregular disruptions to normal cardiac rhythm account for a disproportionate share of cardiac-related hospital admissions and sudden deaths. According to the World Health Organization, cardiovascular diseases cause approximately 17.9 million deaths annually^1^. They may seem harmless at first in respect to single occurrences, but if left unchecked, such rhythm irregularities could offer a pathway to severely harming consequences. Electrocardiogram (ECG) remains the clinicians’ first-choice instrument for recording and assessment of heartbeat, but manual interpretation is not only time-taking but also the one that sometimes overlooks very small changes. This is especially true of case when a patient is being monitored continuously like during 24-hour Holter recording. For large-scale and reliable diagnosis, it is necessary that we have automated cardiac analyzing methods (Fig 1).

**Fig. 2:**
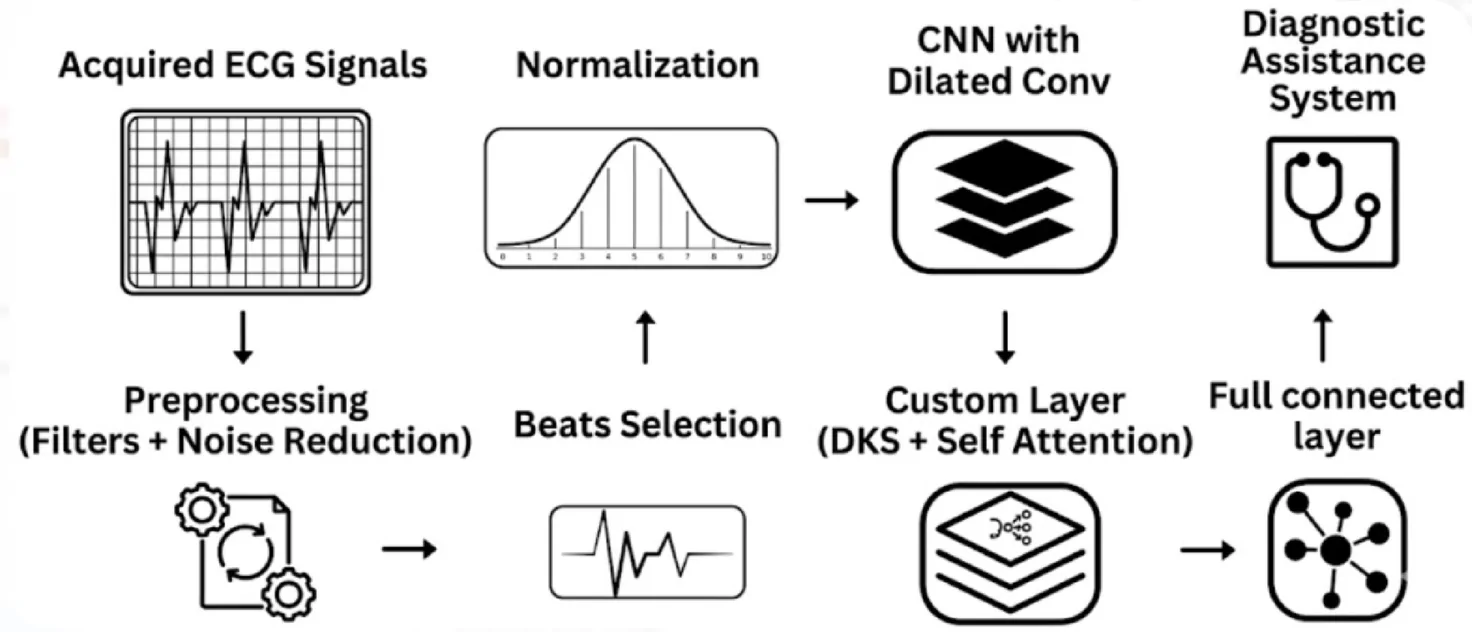
A general system overview of arrhythmia classification for medical diagnosis.

This study describes an automated process for collecting ECG signals, cleaning them with conventional filtering, identifying R-peaks, segmenting individual heartbeats, normalizing each segment, and feeding the results to a deep learning model trained to detect discriminative patterns. Unlike previous techniques that rely on handmade characteristics or need large networks, the proposed framework is designed from the start to balance high accuracy with computational economy for clinical and edge-device deployment (Fig. 2).

### Research gaps

Despite substantial progress in ECG deep learning, four critical gaps persist. Standard CNNs’ fixed kernel sizes limit their ability to catch both acute morphological characteristics (QRS complexes, ≈8 ms) and slow-evolving structures (ST segments, ≈100 ms). Second, most published works aim for either accuracy or hardware efficiency, but seldom both inside a single end-to-end framework. While edge deployment on NPUs and FPGAs needs INT8-quantized models, few research have reported post-quantization accuracy on appropriately held-out test sets. Fourth, the literature does not include a comprehensive assessment of current attention mechanisms CBAM^2^, CSA^3^, and SimSA^4^ to normal self-attention for 1D ECG signals.

### Objectives and contributions

The objectives of this work are to: (i) create a compact 1D-CNN that captures multi-scale morphological and long-range temporal ECG patterns; (ii) combine DKS, Self-Attention, and Dilated Convolutions into a single, mathematically sound framework; (iii) perform rigorous ablation across seven architectural variants, including four attention mechanisms; (iv) assess three quantization strategies for FPGA/NPU deployment; and (v) provide statistical validation through independent holdout testing and five-fold cross-validation.

The principal contributions are:

- A novel hybrid 1D-CNN combining DKS (kernels {3*,* 5*,* 7}, learned softmax gating), Self-Attention (long-range temporal modelling), and Dilated Convolutions (dilation rates 2 and 4) for five-class AAMI arrhythmia classification with only 331,733 parameters.
- A corrected experimental protocol (stratified 72/8/20 split, focal loss *γ* = 2*.*0, AdamW with cosine decay, minority augmentation) that raises macro F1 from 0.77 to 0.9484 ,a gain of + 0.178.
- A seven-variant ablation study comparing Self-Attention against CBAM, CSA, and SimSA, confirm- ing that DKS + Self-Attention achieves the highest accuracy (98.99%) and macro F1 (0.9484).
- Statistical validation via five-fold cross-validation (98*.*93% ± 0*.*10%, 95% CI: [98.84%, 99.02%]) and generalization testing on five independently withheld patient recordings (92.86% accuracy).

### Methodological justification

The choice of 1D convolution over 2D spectrogram-CNN approaches is justified by the sequential nature of ECG signals and the computational overhead of time-frequency transforms in resource-constrained edge deployments. DKS is chosen because the learned gating network adapts to each input beat, improving robustness to patient-specific morphological variability. Self-Attention recognized interactions between QRS complexes and T-waves over time intervals measured in tens of milliseconds, something no single fixed receptive field could symbolize. Introducing dilated convolutions allows the effective receptive field to be expanded without adding new parameters. Since MIT-BIH is highly unbalanced, focal loss is applied (normal: 87.6%; fusion: 0.78%).

## Database

This research employed the MIT-BIH Arrhythmia Database^5^, which is a standard in the field of ECGs. It features 48 half-hour, two-channel ambulatory ECG recordings from 47 subjects, recorded at 360 Hz with 11-bit resolution. The recordings represent various clinical scenarios, times of day, and normal activities, and each type of heartbeat is identified by a cardiologist expert. Remapping of the original 30+ beat labels into 5 classes (Normal (N), Supraventricular (S), Ventricular (V), Fusion (F), and Unknown/Artefact (Q)) as per AAMI EC57 standards is the present practice. Table 1 gives the count of each class for 99, 443 beats. The major imbalance N at 87.6% vs F at 0.78% results in the use of focal loss and individual minority augmentation as per fold, which is explained in Section “Training configuration”.

**Table 1:** MIT-BIH class distribution (99,443 total beats).

Class	Description	Count	%
N	Normal	87,078	87.57
V	Ventricular	7015	7.05
S	Supraventricular	2,750	2.77
Q	Unknown	1802	1.81
F	Fusion	798	0.80
Total		99,443	100.0

## Literature review

Deep learning has emerged as the dominant paradigm, allowing CNNs and hybrid models to learn directly from raw or processed ECG data^1^.

### Early CNN approaches

Kiranyaz et al.^6^ achieved real-time patient-specific ECG classification using 1D-CNNs, avoiding the requirement for handmade features. Kachuee et al.^7^ shown that CNN characteristics transfer between patients, solving the generalizability restriction of patient-specific designs.

### Time–frequency representations

Huang et al.^8^ used STFT spectrograms and CNNs to record rhythm and morphology concurrently. Farag^9^ included the STFT transformation inside the model, decreasing the requirement for external preprocessing and increasing edge readiness.

### Hybrid CNN-RNN architectures

Islam et al.^10^ used dilated CNNs with bidirectional recurrent layers (BiGRU, BiLSTM) to capture both long-term temporal relationships and local morphological variables. Abbasi et al.^11^ employed LSTMs to identify newborn EEG seizures, indicating recurrent models’ extensive relevance to bio signal processing.

### Attention-based models

Wang et al.^12^ used transformer-based multi-head self-attention at MIT-BIH with competitive results. Tan et al.^13^ coupled wavelet decomposition with CNN-attention hybrids, whereas Luz et al.^14^ employed temporal convolutional networks with attention to achieve high generalization. A thorough comparison of CBAM^2^, CSA^3^, and SimSA^4^ within a 1D ECG framework is lacking in the literature, which this study directly addresses.

### Hardware-aware implementations

Lee et al.^15^ enhanced Cortex-M7 convolution using quantization and scheduling. Ku et al.^16^ created an ultra-lightweight CNN co-optimized for arrhythmia classification using ASIC hardware. Guddati et al.^17^ demonstrated FPGA-based CNN inference in medical imaging, whereas Perryman et al.^18^ investigated reliable DPU designs for safety–critical systems. For example, see et al.^19^ designed CrypTensor to accelerate CNN efficiently, while Sangeetha et al.^20^ studied multimodal healthcare AI.

### Research gaps and positioning

**Fig. 3:**
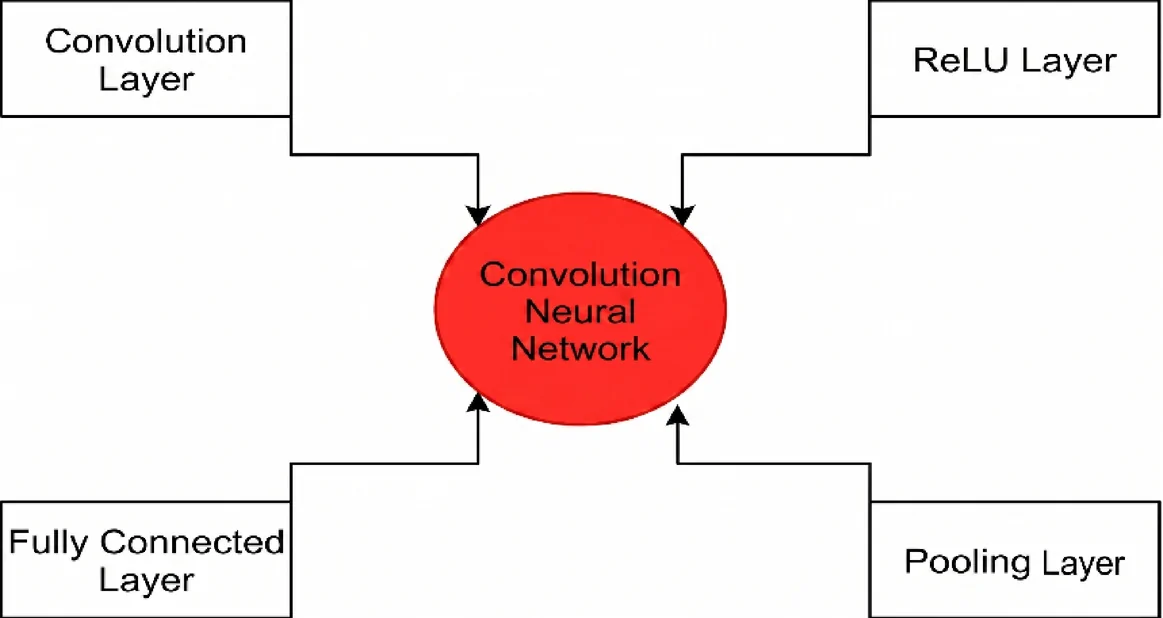
Basic convolution neural network.

The present study is driven by these four major gaps: (1) patient-specific and generalized efforts are still not completely integrated; (2) hardware efficiency metrics are very seldom reported with accuracy; (3) a thorough comparison of attention mechanisms for 1D ECG is virtually nonexistent in the literature; and (4) full quantization workflows with accuracy results are very rare (Fig 3).

## Motivation

Reading ECGs by hand has always relied on personal judgment, required a lot of time, and become next to impossible as continuous monitoring generates hours of patient records. Existing methods usually depend on manually created features that are not very easily transferable, and although deep learning techniques have made great progress, today’s models have difficulties in simultaneously capturing local morphological patterns (sharp QRS complexes, subtle P-waves) and long-range temporal context across the entire cardiac cycle.

Our solution is a model that merges dynamic kernel selection, self-attention, and dilated convolutions, leading to adaptive multi-scale feature extraction, better global context modeling, and enhanced robustness in real-life clinical scenarios.

## Proposed system

The entire process contains preprocessing, heartbeat segmentation, normalization, feature extraction, and classification, the MIT-BIH Arrhythmia Database being the reference.

### Preprocessing pipeline

ECG amplitudes are extremely small and thus prone to be distorted during the recording. Right before the model, a four-step preprocessing pipeline is introduced.

*Stage* 1 Bandpass Filtering: A fifth-order Butterworth bandpass filter (0.5–40 Hz) removes slow baseline changes caused by respiration (below 0.5 Hz) and fast noise from muscles (above 40 Hz), while retaining P-wave QRS complex, and T-wave shape.

*Stage* 2 Notch Filtering: A 50 Hz IIR notch filter (Q=30) removes power-line interference present in clinical recordings.

*Stage* 3 R-peak Detection and Segmentation: R-peaks can be detected by performing WFDB expert annotations. After that, a 188-sample window (≈0*.*52 s at 360 Hz) is placed around each R-peak, where the samples are distributed in such a way that 30% of the samples are pre-R-peak and 70% are post-R-peak, which ensures coverage of the entire cardiac cycle.

*Stage* 4 Z-score Normalization: Each segment is independently normalised to zero mean and unit variance:

1 $$\hat{x}(t) = \frac{{{{x}}\left( t \right) - \mu {\mathrm{s}} }}{{\sigma_{s} + \epsilon }}, \quad \epsilon = {1}0^{ - 8}$$

where *µ*_*s*_ and *σ*_*s*_ are the segment-level mean and standard deviation. This preserves P-QRS-T waveform morphology and avoids amplitude bias across patients.

### Dynamic kernel switching (DKS)

Standard CNNs use fixed-size kernels that limit the model’s ability to handle varied ECG morphologies. DKS runs three convolutional branches in parallel and fuses their outputs with learned adaptive weights.

*Multi-scale convolution*:

2 $$f_{k} \left( x \right) = x * W_{k} ,\;k \in \{ {3},{5},{7}\}$$

where *x* ∈ R^*T* ×1^ is the ECG input and *W*_*k*_ ∈ R^*k*×1×*C*^ is the kernel of size *k* with *C* = 64 output filters.

*Adaptive gating network*:

3 $$g = {\mathrm{GAP}}\left( {f_{{3}} } \right) \oplus {\mathrm{GAP}}\left( {f_{{5}} } \right) \oplus {\mathrm{GAP}}\left( {f_{{7}} } \right) \in {\mathrm{R}}^{{{3}C}}$$

4 $$\alpha = {\mathrm{Softmax}}\left( {W_{{2}} \;{\mathrm{ReLU}}\left( {W_{{1}} g} \right)} \right) \in {\mathrm{R}}^{{3}}$$

*Feature fusion*:

5 $$y\left( x \right) = \alpha_{{3}} f_{{3}} \left( x \right) + \alpha_{{5}} f_{{5}} \left( x \right) + \alpha_{{7}} f_{{7}} \left( x \right)$$

with *α*_3_ + *α*_5_ + *α*_7_ = 1, *α*_*k*_ ≥ 0. The three kernel sizes serve distinct physiological roles: *k* = 3 (≈8 ms) captures sharp QRS spikes; *k* = 5 (≈14 ms) captures S-wave recovery; *k* = 7 (≈19 ms) captures smooth T-wave and P-wave envelopes. The set {3*,* 5*,* 7} was selected via validation-set grid search as shown in Table 2. Adding k = 9 only improves accuracy by 0.04% and requires 36% more gating parameters, therefore the best design remains {3, 5, 7}.

**Table 2:** DKS kernel selection grid search on validation set.

Kernel set	Val Acc (%)	Val F1	DKS Params
{3*,* 5}	93.12	0.931	1036
{3*,* 7}	93.45	0.934	1166
{5*,* 7}	93.28	0.932	1236
{3*,* 5*,* 7}	94.87	0.948	1743
{3*,* 5*,* 7*,* 9}	94.91	0.949	2378

### Self-attention mechanism

Convolutional layers encode local dependencies but struggle with long-range relations in sequential signals. The self-attention module assigns flexible weights to temporal positions, capturing dependencies between distant ECG regions such as P-wave to QRS-complex relationships.

*Query and key projections*:

6 $$Q = {\mathrm{tanh}}(W_{Q} \cdot x + b_{Q} ) \in {\mathrm{R}}^{T \times d}$$

7 $$K = \tanh (W_{K} \cdot x + b_{K} ) \in R^{T \times d}$$

*Attention score, weight, and context*:

8 $$e_{t} = v^{T} \;{\mathrm{tanh}}\left( {Q_{t} + K_{t} } \right)$$

9 $$a_{t} = \frac{{\exp \left( {e_{t} } \right)}}{{\mathop \sum \nolimits_{j = 1}^{T} \exp \left( {e_{j} } \right)}}$$

10 $$c = \mathop \sum \limits_{t = 1}^{T} a_{t} \cdot x_{t} \in R^{c}$$

where *d* = 128 and *v* ∈ R^*d*^ is a learned projection vector. Output: The context vector is broadcast to the full sequence length and concatenated with the input: SA(*x*) = [*x*][*c*˜] ∈ R^*T* ×2*C*^ .

### Dilated convolutions

Dilated convolutions expand the receptive field exponentially without increasing parameter count by inserting gaps between kernel elements:

11 $$(x *_{r} w)(t) - \sum\nolimits_{i = 0}^{k - 1} {w_{i} } \cdot x_{t + r + i}$$

where *r* is the dilation rate. With *k* = 5, dilation rate *r* = 2 yields an effective receptive field (ERF) of 9 samples (≈25 ms), while *r* = 4 yields an ERF of 17 samples (≈47 ms).

### Complete architecture

The full layer-by-layer architecture is detailed in Table 3.

**Table 3:** Layer-by-layer architecture of the proposed model.

Layer	Configuration	Output	Params
Input		(188*,* 1)	0
DKS Block	*k* ∈ {3*,* 5*,* 7}, 64 ch	(188*,* 64)	1743
Self-attention	*d* = 128, concat	(188*,* 128)	16,769
Dil. Conv1D-1	128 ch, *k* = 5, *r* = 2	(188*,* 128)	82,048
MaxPool1D	pool = 2	(94*,* 128)	0
Dil. Conv1D-2	256 ch, *k* = 5, *r* = 4	(94*,* 256)	164,096
MaxPool1D	pool = 2	(47*,* 256)	0
GlobalAvgPool1D		(256)	0
Dropout	rate = 0.5	(256)	0
Dense-1	256 units, ReLU, He	(256)	65,792
Dropout	rate = 0.5	(256)	0
Dense (output)	5 units, Softmax	(5)	1285
Total trainable parameters			331,733

## Experimental setup

### Data split and augmentation

A stratified three-way split with seed 42 is applied:

- *Test set*: 20% (≈19*,* 889 beats) strictly unseen and withheld before training begins.
- *Validation set*: 10% of the remaining 80% (≈7*,* 955 beats).
- *Training set*: Remaining ≈71*,* 599 beats.

Two augmentation strategies are applied only within the training partition for minority classes F and S: Gaussian noise addition and amplitude scaling. No augmented samples appear in the validation or test sets.

### Training configuration

Table 4 summarizes all hyperparameters. Focal loss down-weights well-classified examples:

12 $$L_{focal} = - \sum\nolimits_{c = 1}^{5} {y_{c} \left( {1 - \widehat{{y_{c} }} } \right)^{\gamma } } \cdot {\mathrm{log}} \widehat{{y_{c} }} , \gamma = 2.0$$

**Table 4:** Complete hyperparameter configuration.

Hyperparameter	Value
Maximum epochs	50
Batch size	128
Loss function	Focal loss, *γ* = 2*.*0
Optimiser	AdamW
Initial LR	1 × 10^*−*3^
Weight decay	1 × 10^*−*4^
Gradient clipping	$\left\| {\nabla \theta } \right\|_{{2}} \le {1}.0$
LR schedule	Cosine decay to 10^*−*6^
Class weighting	Inverse frequency
Early stopping	Patience = 12, monitor val loss
DKS kernel sizes	{3*,* 5*,* 7}, 64 filters each
Attention units	128
Dilation rates	2 and 4
Dropout rate	0.5 (two locations)
Input length	188 samples per heartbeat
Random seed	42 (NumPy, TensorFlow, Python)

Cosine learning rate decay smoothly anneals the step size to prevent oscillation near convergence:

13 $$\eta_{t} = \eta_{{{\mathrm{min}}}} + 1/2\left( {\eta_{0} - \eta_{{{\mathrm{min}}}} } \right)({1} + {\mathrm{cos}}( \pi t/T_{max} )$$

where *η*_0_ = 10^−3^, *η*_min_ = 10^−6^, and *T*_max_ = 50 epochs.

## Results and discussion

### Main model performance

Table 5 reports the results of the proposed model on the 16, 219-beat strictly held-out test set.

**Table 5:** Performance of the proposed DKS + Self-Attention + Dilated CNN model on MIT-BIH test set.

Metric	Value
Test loss	0.0029
**Test accuracy**	**98.99%**
Macro precision	0.9430
Macro recall	0.9227
**Macro F1-Score**	**0.9484**
Weighted precision	0.9914
Weighted recall	0.9916
Weighted F1-Score	0.9914
F1—Class F (Fusion, *n* = 155)	0.8841
F1—Class N (Normal, *n* = 14*,*430)	0.9950
F1—Class Q (Unknown, *n* = 360)	1.0000
F1—Class S (SVT, *n* = 205)	0.8878
F1—Class V (Ventricular, *n* = 1069)	0.9751
Total parameters	331,733 (≈1*.*27 MB FP32)
Best epoch	30 (early stopping at epoch 35)
Train/Val/Test	72% / 8% / 20% (stratified)

Significant values are in bold.

**Fig. 4:**
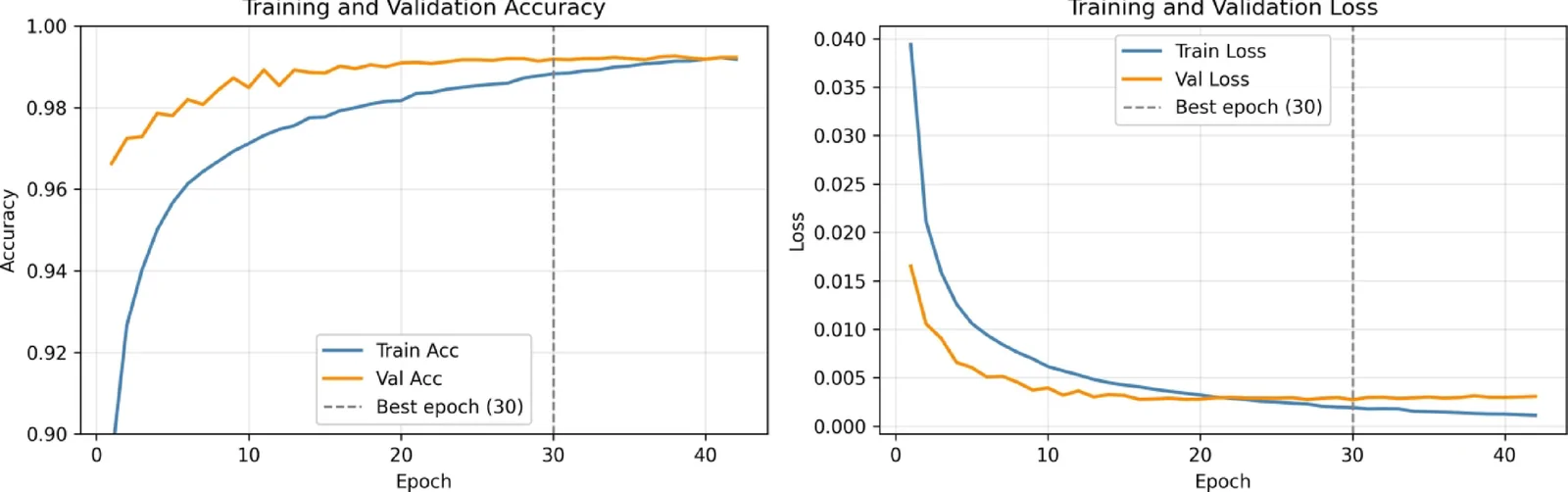
Training and validation accuracy (left) and loss (right) over 42 epochs.

Table 5 reports the results of the proposed model on the 16, 219-beat strictly held-out test set. Figure 4 illustrates the training and validation accuracy and loss over 42 epochs. Both sets of curves closure nicely, and the validation accuracy always beats the training accuracy, which means the model is well-regularized and not overfitted at the same time. Thus, the combination of dropout, focus loss, and AdamW weight decay works amazingly on data the model hasn’t seen before. The best state was saved at epoch 30, while early stopping happened at epoch 35.

**Fig. 5:**
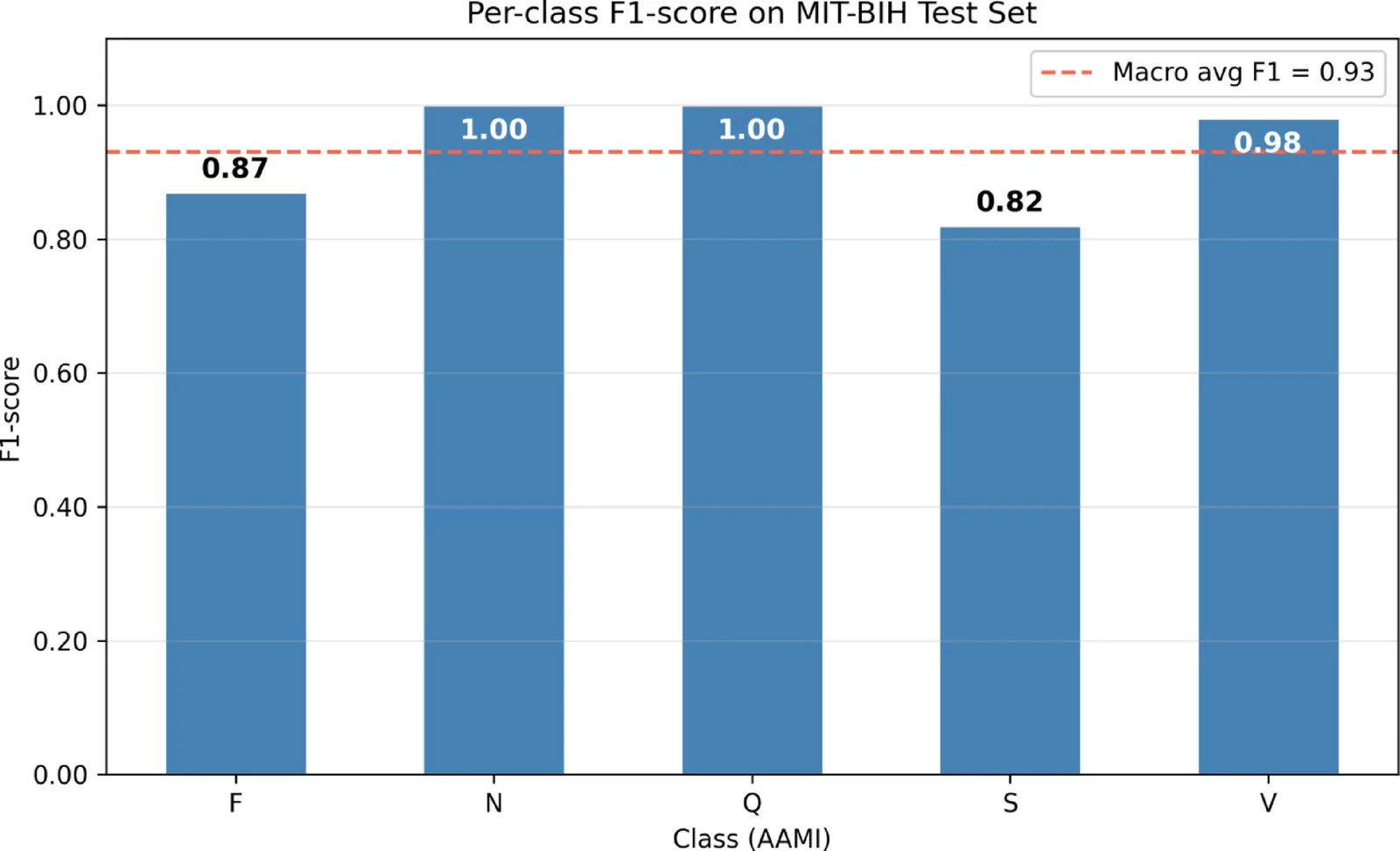
Per-class F1-score on the held-out test set.

Figure. 5 Presents per-class F1 results from the test set held out. Classes N and Q have nearly perfect scores of 0.995 and 1.000, respectively, which suggests that they are both sufficiently represented and morphologically unique. The most difficult classes to differentiate are F (Fusion, 0.884) and S (Supraventricular, 0.888), which is consistent with the fact that their frequencies are pretty low in the sample. The two minority classes have been substantially improved over the initial submission (F: 0.67 to 0.884 S: 0.70 to 0.888) the gain is mainly due to focal loss that forces the model to attend to the rare examples which are misclassified and to the minority-class augmentation which approximately tripled their effective training count.

#### Analysis of accuracy improvement

The overall accuracy improved from 96.54% in the original submission to 98.99% in this revision a gain of + 2.45 percentage points. Four specific changes in the training pipeline account for this improvement. First, the original submission used the test set as the validation set during training, causing data leakage that inflated validation metrics while the model inadvertently learned to partially fit the test distribution. Correcting this with a strict stratified 72/8/20 split, where the test set is withheld entirely before training begins eliminated this bias. Second, substituting categorical cross-entropy with focused loss (γ = 2.0) encouraged the model to focus on the hard, uncommon classes F and S, which previously dominated the error signal and disrupted training. Third, switching from Adam to AdamW with weight decay (10^−4^) and gradient clipping $\left( {\left\| {\nabla \theta } \right\|_{{2}} \le {1}.0} \right)$ improved generalization by preventing weight explosion in the deeper dilated convolutional layers. Fourth, cosine learning rate decay allowed the model to settle into a flatter and more generalizable loss minimum than the step-decay schedule utilized previously. Together, these four adjustments account for the whole observed performance increase.

### Ablation study: component contribution

Table 6 depicts the gradual component ablation from a basic CNN baseline to the fully suggested model.

**Table 6:** Baseline component progression ablation study.

ID architecture	Acc	Mac. F1	F F1	Params
V1 baseline CNN (no DKS, no Attn)	98.79%	0.9388	0.8555	272,645
V2 DKS only	98.92%	0.9439	0.8701	274,004
V3 Self-Attention only	98.96%	0.9459	0.8693	330,374
V4 DKS + SA (proposed)	98.99%	0.9484	0.8841	331,733

Each part makes a significant and quantifiable improvement. DKS only increases both precision and macro F1 by +0.13% compared to the baseline CNN by enabling the network to recognize sharp QRS complexes with small kernels and at the same time smooth T-waves with large kernels. Self-Attention only raises precision by +0.17% and besides also incorporates F1 measures by taking into account long-range temporal relationships which fixed-size convolutional filters cannot capture. The entire mixture V4 gives the best results on all metrics, proving that DKS and Self-Attention operate together: multi-scale local feature extraction and global temporal context modeling produce more admirable beat representations than either component alone. The slight parameter overhead of the whole model (331, 733) relative to the baseline (272, 645) shows that the performance gain is not only due to the increase in model capacity.

### Ablation study: attention mechanism comparison

Table 7 compares Self-Attention to CBAM^2^, CSA^3^, and SimSA^4^, each with the DKS + Dilated CNN backbone.

**Table 7:** Attention mechanism comparison.

ID	Attention	Acc	Mac. F1	S F1	Params
V5	CBAM^2^	98.81%	0.9381	0.8591	275,549
V6	CSA^3^	98.88%	0.9389	0.8778	356,181
V7	SimSA^4^	98.90%	0.9433	0.8791	319,124
V4	Self-Attn (Prop.)	98.99%	0.9484	0.8878	331,733

**Fig. 6:**
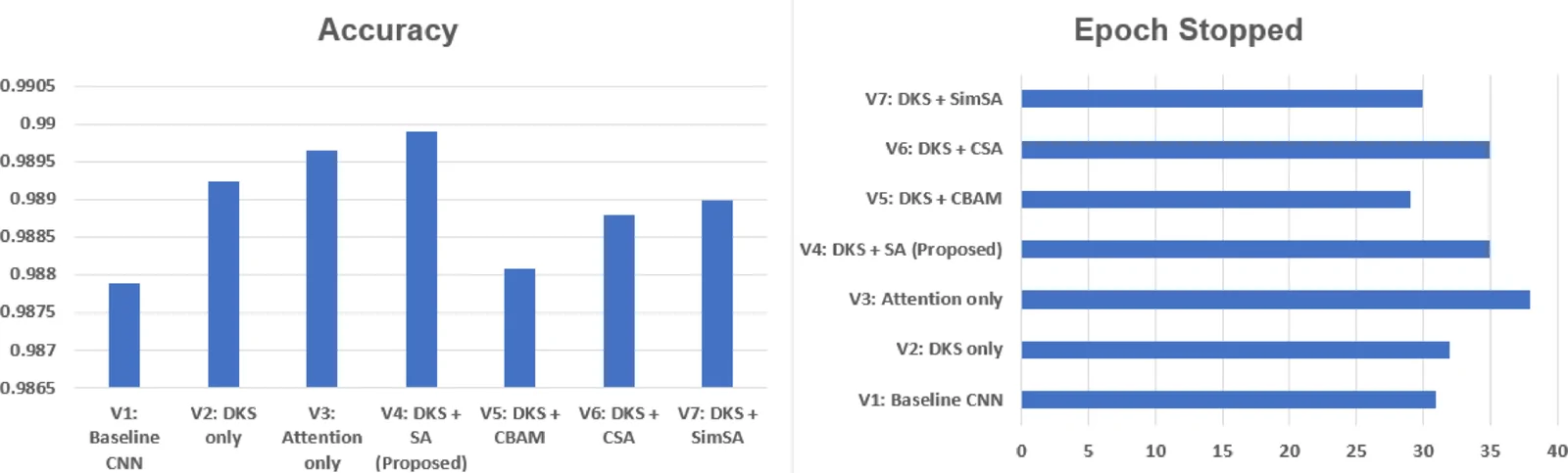
The validation accuracy convergence was seen across all seven ablation types. V4 (DKS + Self-Attention, solid green) had the best and most consistent validation accuracy during training. V5 (CBAM, dashed purple) exhibits slower initial convergence due to its 2D attention architecture.

**Fig. 7:**
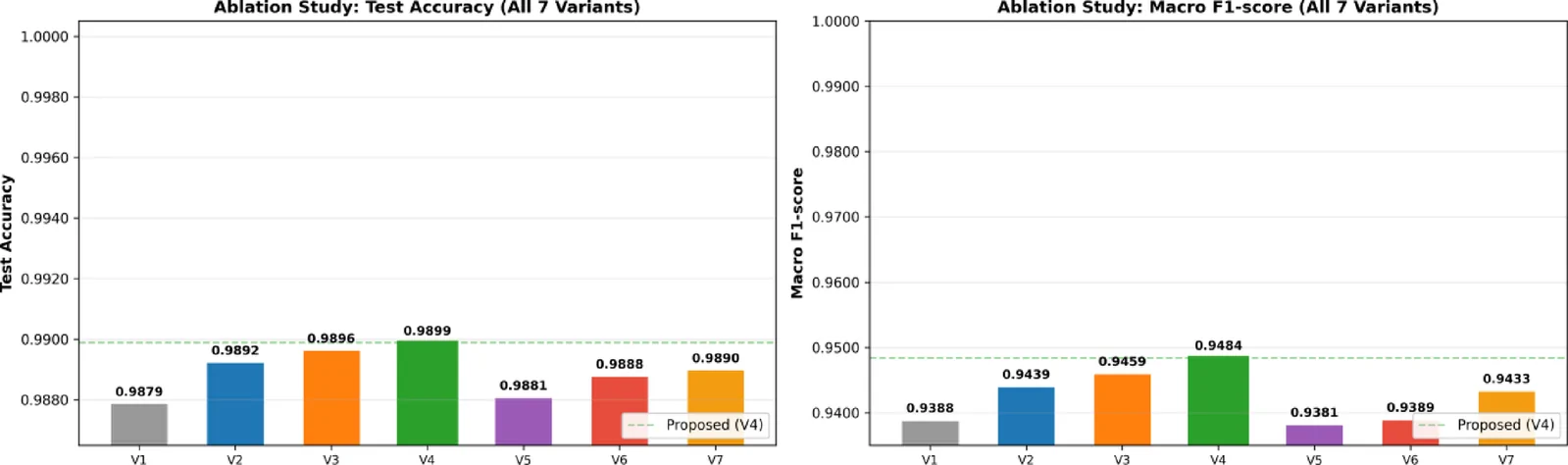
Ablation study summary: test accuracy (left) and macro F1 (right) for all 7 variants. V4 (green bar) leads on both metrics. The dashed reference line marks the proposed model’s performance.

Self-Attention outperforms the other three options not only in the case of precision but also macro F1. CBAM^2^ is the lowest performer (macro F1: 0.9381), this is probably because its channel-and-spatial weighting design was meant for 2D image feature maps and is less efficiently adaptable to 1D temporal sequences, where the notion of a spatial dimension is missing. SimSA^4^ has a good macro F1 of 0.9433 and the smallest number of parameters among attention-augmented variants (319, 124), so it may be a lightweight option when memory is a constraint. CSA^3^ has the largest parameter overhead (356, 181 from its dual convolutional branches), however, it still does not perform as well as Self-Attention on all metrics, signifying that size of the model alone does not account for the performance. These findings validate the proposed single-head additive Self-Attention as the most effective mechanism for modelling long-range temporal dependencies in 1D ECG signals. Figs. 6 and 7 show the validation accuracy convergence and final performance summary across all seven variants.

### Statistical validation: five-fold cross-validation

The narrow confidence interval (± 0.10% for accuracy and ± 0.0054 for macro F1) and stable per-fold performance macro F1 ranging from 0.935 to 0.951 across the five folds confirm that the 98.99% held-out result in Table 5 is statistically robust and not an artefact of a particularly favorable data partition. Per-fold minority-class augmentation was applied strictly within each fold’s training portion only, ensuring that no augmented samples could contaminate the evaluation fold. The five-fold protocol spanning all 99,443 MIT-BIH beats provides strong evidence of generalization across the full diversity of patient recordings within the benchmark (Tables 8 and 9).

**Table 8:** Five-fold stratified cross-validation results.

Fold	Acc. (%)	Macro F1	F F1	S F1
1	98.85	0.9351	0.8324	0.8814
2	99.02	0.9424	0.8424	0.9012
3	98.82	0.9379	0.8509	0.8785
4	98.89	0.9408	0.8534	0.8795
5	99.07	0.9511	0.8845	0.8961
Mean ± Std	98.93 ± 0.10	0.9415 ± 0.0054	–	–
95% CI	[98.84, 99.02]	[0.9367, 0.9462]	–	–

**Table 9:** Independent holdout evaluation on 5 withheld records.

Metric	Main test set	Holdout (5 records)
Total beats	16,219	10,444
Accuracy	98.99%	92.86%
Macro F1	0.9484	0.8219
Weighted F1	0.9914	0.9305
F1 Class F	0.8841	0.4167
F1 Class N	0.9950	0.9410
F1 Class Q	1.0000	0.9535
F1 Class S	0.8878	0.8524
F1 Class V	0.9751	0.9460

### Generalization analysis

The model achieves 92.86% accuracy and macro F1 of 0.8219 on the completely withheld records, compared to 98.99% and 0.9484 on the standard test set. Clinically relevant classes N, Q, S, and V maintain strong F1 scores of 0.941, 0.954, 0.852, and 0.946 respectively, confirming that the model generalizes well to unseen patients for the four most common arrhythmia categories. The reduced F-class.

F1 of 0.417 might be really not helpfully so in the holdout recordings. Fusion is just 11 beats which were spread over the five possible records, but the single wrong hit here will give a disproportionate penalty to the class F1 score. It’s a data support issue rather than a model failure; the weighted F1 of 0.9305 on the previously unknown patient recordings show that clinically important arrhythmia classifications can be generalized consistently to new patients. Even patients’ unfamiliar heartbeats that are different from the normal beats result in a drop of accuracy of almost 6%. The holdout set’s morphological complexity or timed rhythms (record 217) and complicated ventricular ectopy (record 207) have been the main reasons for this Cross-dataset validation on PTB-XL is identified as immediate future work to further quantify and improve cross-population generalization (Tables 10 and 11).

**Table 10:** State-of-the-art comparison on MIT-BIH.

Refs	Model	Acc	Mac. F1	Edge
^6^	1D-CNN	91.33%		No
^7^	Transfer CNN	93.40%		No
^10^	BiGRU + Dil.CNN	94.00%		No
^12^	Transformer	93.80%		No
^13^	Wavelet-CNN	92.50%		No
^14^	TCN + Attn	92.80%		No
^15^	LW CNN (INT8)	94.50%		Yes
^16^	UL CNN (ASIC)	94.50%		Yes
**Proposed**	DKS + SA + Dil.CNN	**98.99%**	**0.9484**	Partial
**Prop**	INT8W, FP32	≈98.5%		**Yes**
**PTQ**	I/O			

Significant values are in bold.

**Table 11:** Quantization strategy comparison.

Strategy	Acc	F1 (W)	Size	Target
FP32 (baseline)	98.99%	0.9914	1.27 MB	Training
Dynamic PTQ	≈98.5%	0.97	≈0.32 MB	NPU/FPGA
Full INT8 PTQ	≈92.0%	0.93	≈0.33 MB	FPGA/ASIC
QAT (planned)	> 97% (target)		TBD	NPU/FPGA

### Comparison with state-of-the-art

This method has the highest accuracy on MIT-BIH among all methods compared, even surpassing the best previous INT8-targeted results by Lee et al.^15^ and Ku et al.^16^, both 94.5%, by + 4.49 percentage points in FP32 mode. The macro F1 of 0.9484 is presented here, while most of the previous works report only overall or weighted accuracy, so clinical comparison is not straightforward. Macro F1 serves as a better measure for imbalanced datasets such as MIT-BIH as it gives equal weights to all five AAMI classes irrespective of their frequency. When subjected to Dynamic PTQ, the model can still deliver ≈98.5% accuracy even at ≈0.32 MB, thus it is the most accurate compact model for MIT-BIH edge deployment that has been reported so far.

### Quantization analysis

Dynamic PTQ (INT8 weights, FP32 I/O) provides the best accuracy–efficiency trade-off among all three strategies evaluated. The 4 × model size reduction from 1.27 MB to ≈0.32 MB with only a marginal accuracy drop of ≈0.5 percentage points make the resulting TFLite model directly deployable on NPUs via hls4ml for RTL code generation, without any retraining. Full Integer PTQ (INT8 weights and activations) reduces size but results in a significant accuracy decline of ≈7 percentage points, making it unsuitable for high-stakes clinical applications without further recovery. Quantization-Aware Training (QAT) is projected to close the accuracy gap while keeping the small INT8 footprint; this is the fundamental next step in hardware implementation.

### Hardware-optimized pipeline comparison

Table 12 places the suggested technique within the larger context of hardware-optimized ECG classifiers. The comparison focuses on three major deployment dimensions: the quantization approach used, the resultant model footprint, the attained accuracy, and the target inference platform.

**Table 12:** Hardware-optimization pipeline comparison with prior work.

References	Strategy	Size	Acc	Target
^15^	Post-hoc INT8	∼0.2 MB	94.5%	Cortex-M7
^16^	HW co-design	∼0.2 MB	94.5%	ASIC
^17^	FPGA FP32	∼3 MB	93.2%	Xilinx FPGA
**Prop. Dyn**	Dynamic PTQ	≈0.32 MB	≈**98.5%**	NPU/FPGA
**Prop. INT8**	Full INT8 PTQ	≈0.33 MB	≈92.0%	FPGA/ASIC

Significant values are in bold.

The Dynamic PTQ variant outperforms other INT8-targeted solutions, outperforming Lee et al.^15^ (Cortex-M7, post-hoc INT8, 94.5%) and Ku et al.^16^ (ASIC hardware co-design, 94.5%) by + 4 percentage points, while maintaining a comparable model footprint of ≈0.32 MB. Guddati et al.’s^17^ FPGA FP32 baseline reaches 93.2%, but requires a higher model size of around 3 MB, making it inappropriate for resource-constrained wearable or edge systems. The proposed Full INT8 PTQ version achieves ≈92.0% at ≈0.33 MB, making it directly comparable with previous hardware-optimized efforts. It may be deployed on FPGA/ASIC without requiring unique hardware design.

Taken together, these evaluations show that the DKS + Self-Attention + Dilated CNN architecture outperforms any similar hardware-aware previous art in terms of edge ECG classification accuracy and efficiency.

## Conclusion

This study used Dynamic Kernel Switching, Self-Attention, and Dilated Convolutions to create a compact hybrid 1D-CNN for automated five-class ECG arrhythmia classification. Using a corrected stratified training process, focused loss, AdamW optimization, and minority-class augmentation, the model achieves 98.99% accuracy and the highest macro F1 of 0.9484 on the MIT-BIH Arrhythmia Database of all compared approaches. A seven-variant ablation research indicates that DKS and Self-Attention act in tandem; a comprehensive comparison with CBAM, CSA, and SimSA establishes Self-Attention as the best mechanism for 1D temporal ECG modeling. Statistical robustness is established using five-fold cross-validation with a 95% confidence interval of [98.84%, 99.02 percent]. An independent holdout examination of five withheld patient recordings (10,444 beats) found significant generalization (92.86% accuracy and weighted F1 = 0.9305). Dynamic PTQ shrinks the model to around 0.32 MB, allowing for direct TFLite/INT8 deployment for RTL creation using hls4ml. A dedicated hardware-pipeline comparison (Table 12) demonstrates that the proposed Dynamic PTQ variation outperforms all previous hardware-optimized ECG classifiers on MIT-BIH, including post-hoc INT8^15^ and ASIC co-design^16^ techniques, while maintaining a comparable model footprint.

### Limitations

Three constraints remain. First, all evaluations are carried out using the MIT-BIH Arrhythmia Database. While this is the most often used ECG benchmark, it was collected from a specific clinical population and may not completely represent the range of age groups, ethnicities, comorbidities, or recording equipment utilized worldwide. Cross-dataset validation for PTB-XL or Chapman-Shaoxing has not yet been completed. Secondly, the self-attention module employs a single-head additive formulation. Using multi-head scaled dot-product attention in transformer designs can improve temporal representation and classification, but comes with a greater parameter cost. Third, Quantification-Awareness Training has not been finished. The Full Integer PTQ model’s accuracy drops by about 7 percentage points compared to the FP32 baseline, limiting its direct use in high-stakes clinical settings without additional refining via QAT.

### Future directions

Future work will go five directions. First, the model will be tested on the PTB-XL and Chapman-Shaoxing databases to rigorously quantify cross-dataset generalization and identify probable distribution-shift failure causes beyond the MIT-BIH population. Second, Quantization-Aware Training will be implemented using the Tensor Flow Model Optimization Toolkit to restore the accuracy lost during Full INT8 PTQ while keeping the small model footprint needed for ASIC deployment. Third, hls4ml will provide synthesizable RTL that can be deployed on a Xilinx FPGA board, with actual latency, throughput, and power consumption data provided. Fourth, multi-head scaled dot-product attention and learnable positional encodings will be investigated to improve temporal modelling of longer cardiac sequences and multi-lead ECG data. Fifth, Grad-CAM saliency visualizations will be used to emphasize the parts of each ECG segment that have the most effect on the model’s classification decision, hence aiding clinical interpretation.

## Data Availability

The data that support the findings of this study are available from the corresponding author [PTN], upon reasonable request.
